# Epigenome-Wide Association Study Reveals Differential Methylation Sites and Association of Gene Expression Regulation with Ischemic Moyamoya Disease in Adults

**DOI:** 10.1155/2022/7192060

**Published:** 2022-03-24

**Authors:** Shihao He, Xun Ye, Ran Duan, Yahui Zhao, Yanchang Wei, Yanru Wang, Ziqi Liu, Xiaokuan Hao, Xiaolin Chen, Qiang Hao, Hao Wang, Yuanli Zhao, Rong Wang

**Affiliations:** ^1^Department of Neurosurgery, Beijing Tiantan Hospital, Capital Medical University, Beijing 10070, China; ^2^Department of Neurosurgery, Peking University International Hospital, Beijing 102206, China; ^3^Center of Stroke, Beijing Institute for Brain Disorders, Beijing 100069, China

## Abstract

**Background:**

The association of DNA methylation with the pathogenesis of adult ischemic moyamoya disease (MMD) is unknown. Here, we investigated the genome-wide DNA methylation profiles in patients with MMD and identified the genes related to the pathogenesis of MMD.

**Methods:**

Whole blood samples were collected from 20 individuals, including 10 patients with ischemic moyamoya disease without any underlying disease and 10 healthy individuals. Genome-wide DNA methylation analysis was performed using Illumina 850K microarrays. Transcriptional correlation was verified using quantitative reverse transcription-polymerase chain reaction. In vitro experiments were used to analyze the association of functional defects with candidate epigenetic markers.

**Results:**

The genome-wide methylation level in the whole blood of adults with ischemic MMD was higher than that in the healthy individuals. In total, 759 methylation probes differed significantly between the case and control. The hypermethylated regions were mostly concentrated in the gene spacer regions. Among genes with the highest degree of the differential expression, KCNMA1 and GALNT2 were upregulated, whereas SOX6 and RBM33 were downregulated.

**Conclusions:**

This is the first study showing that the low expression of genes associated with epigenetic regulation, such as SOX6 and RBM33, may be related to vascular occlusion in MMD, whereas the overexpression of KCNMA1 and GALNT2 may be related to the vascular hyperplasia. The results suggest that DNA methylation was involved in the pathogenesis of MMD, and new pathogenic genes were proposed as biological markers.

## 1. Background

Moyamoya disease (MMD) is a rare chronic disease of unknown etiology characterized by progressive stenosis of the internal carotid artery and a “smoky” abnormal vascular network in the brain tissue at the base of the skull (1). In recent years, because of the continuous innovation in high-throughput sequencing technology, more putative susceptibility genes related to MMD have been identified. These include the hot spot genes RNF213, DIAPH1, and ACTA2, which have been shown to be closely associated with various vascular diseases (2–5). These studies have shed light on the complexity of the mechanisms involved in the development of MMD.

At present, the effect of the RNF213 mutation on MMD is still hypothetical. In a study using a mouse model to knock out the gene, there were no significant changes in the anatomy of the circle of Willis of either the homozygous RNF213-KI or the wild-type mice. Even after ligation of the carotid artery in mice, there was no remodeling of intracranial vessels (6). A recent study on the factors influencing polygenic variation in MMD revealed that the pathogenesis was closely related to the mutual crosstalk among multiple genes, loci, pathways, and functions (7). Therefore, in addition to susceptibility genes, epigenetic regulation and environmental factors may lead to the occurrence of MMD.

In a study of DNA methylation in endothelial colony-forming cells from three patients with MMD, The sortilin1 overexpression was found to regulate the expression of major angiogenic factors and matrix metalloproteinase-9 (8). However, differences in DNA methylation levels of whole blood genetic material between patients with MMD and healthy controls and their effects on the pathogenesis of ischemic MMD have not been studied so far.

Here, we aimed to understand the role of differential DNA methylation in the pathogenesis of ischemic MMD in adults. Cell proliferation, EdU, flow cytometry, and tubule formation assays were performed on genes both upregulated and downregulated by methylation. We propose that the associated genes regulated by DNA methylation may be an important factor for angiogenesis in MMD and development of the disease.

## 2. Results

### 2.1. Whole DNA Methylation Profile in Patients with MMD

The clinical data of the patients are described in Table 1. To elucidate the genome-wide methylation distribution in patients with MMD and its differences with that in normal controls, we used the Illumina Human Methylation EPIC microarray. DNA methylation analysis was performed on whole blood samples from adults with ischemic MMD (*n* = 10). The original probe data of 20 samples were quality-controlled and pretreated. In total, 726,339 probe sites were used for further analysis. To comprehensively analyze the methylation level in all samples, we performed unsupervised hierarchical clustering for all probes (*n* = 726339). DNA methylation followed an equilibrium distribution pattern in all samples, and DNA methylation levels differed in most of the MMD patient groups (>80% and <20%). The distribution of specific gene regions with DNA methylation was similar in the patients and healthy control individuals. Genome-wide methylation analysis revealed a greater methylation tendency in the MMD group compared to the healthy control group, which was characterized by a greater abundance of significant methylation sites or a higher degree of methylation at the same sites in the MMD group. The median DNA methylation of gene promoters was higher than that of the normal controls.

### 2.2. Quality Control of the Sequencing Results of Differentially Methylated Sites and Results of Whole Gene Association Analysis

**Figure 1:**
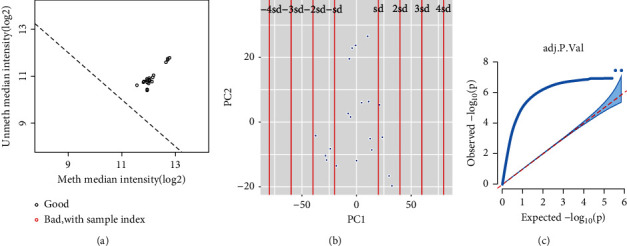
(a) Analysis of overall signal median value. The abscissa shows the median signal value of the methylation site in the sample (log 2), and the ordinate shows the median signal value of the non-methylation site in the sample (log 2). All samples are represented as scattered points. There were no outliers in the adult samples of ischemic moyamoya disease and normal control samples. (b) Principal component analysis (PCA). After screening and filtering all differentially methylated sites, PCA was performed on all samples. All samples appear as scattered points in the first, second, and third principal component spaces. All samples are in the range of 4 standard deviations. ±1 sd-4 sd represent the four times standard deviation range, and the horizontal and vertical coordinates represent the acceptance range of principal component 1 and principal component 2, respectively. (c) CpG site association analysis and quantile-quantile map. Genome-wide association analysis was performed for all CpG loci with differential methylation. Significant observed values after adjustment for all sites were presented in the figure in the form of scattered points. As shown here, the pleiotropy of target genes at different methylation sites was significant. The horizontal and vertical coordinates represent the expected value loci and the actual value loci, respectively, of the results of CpG-whole gene association analysis.

The different methylation sites detected using the 850K chip were summarized and sorted, and multiple data quality controls were performed to improve the stability and reliability of the data. The dual probe technology unique to the 850K chip (Infinium I and Infinium II) extends the detection range to 726,339 probe sites. The methylation site signals at the CpG site were basically similar to those at the nonmethylation site, and the median of the methylation site signals was slightly higher than that of the nonmethylation site signals (Figure 1(a)). This proves that all detected CpG loci, whether significant or not, have been taken into account before data pretreatment and analysis. According to the sample distribution, all the samples were in the first and second principal component dimensions and within two times standard deviation (Figure 1(b)). On this principal component dimension, all our samples should be subjected to further processing and analysis. As the samples tested well, the credibility of our methylation profile analysis in the case group and the control group was further enhanced. According to the results of the whole gene association analysis of CpG loci, our samples showed obvious gene pleiotropy (Figure 1(c)). As we were screening samples from a population, those with high frequency of SNP loci (1986) could be deleted; at the same time, to avoid gender bias, we also removed the CpG loci on sex chromosomes from our analysis. Importantly, the samples were from a single population from the little yellow Han plain area in East Asia.  Table 2 shows the top ten CPGs with the highest differential expression.

### 2.3. Target Gene Pathway Enrichment and Significant Expression at Different Methylation Sites

**Figure 2:**
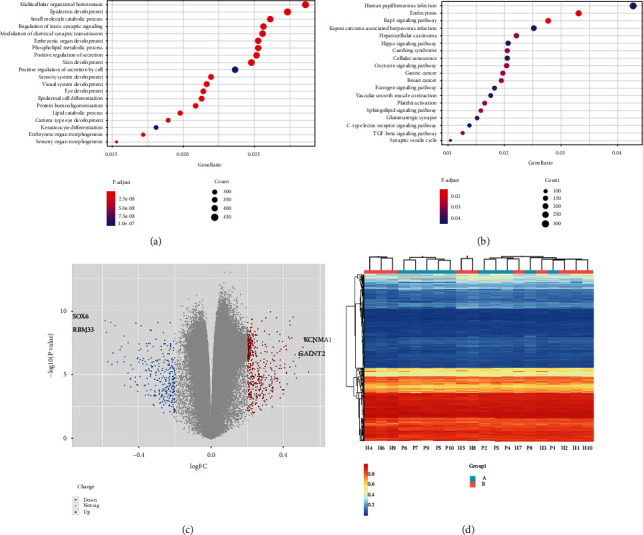
(a) The annotation results of all the different target genes were sorted according to the adjusted enrichment degree (*P*. adjust) and the number of overlapping genes (count). (b) The KEGG database was used to show the pathways associated with differentially methylated genes, and the rankings were also based on *P*. adjust and count. The size of the scatter represents the number of corresponding genes (count), and the closer the color is to red, the stronger is the significance (*P*. adjust). (c) The target gene scatter-volcano map of differentially methylated gene loci; the blue dots on the upper left and the red dots on the upper right of the volcano map indicate that the changes in expression are more than 1 (FC ≥ 1, *P* value <0.5), the downregulation and upregulation trends. Among them, there are 758 red dots and 256 blue dots. (d) The heat map shows all the different methylation sites in the 20 samples. The dark color denotes the hypermethylation site, and the light color denotes the hypomethylation site.

For determining the distribution of the target genes at different methylation sites, we focused on the interaction among different target genes. The molecular biological functions of the nearby genes involved in the same biological function will be discussed further. These genes in the differentially methylated regions were annotated using Gene Ontology (GO) analysis. An enrichment analysis of the functional enrichment analysis of the genes in the differentially methylated region is shown in Figure 2(a). We performed a comprehensive cluster analysis of the key biological pathways such as signal transduction and cell metabolism pathways of the differentially methylated genes (Figure 2(b)). Interestingly, in the GO cluster analysis of molecular biology, “multicellular organismal homeostasis,” “skin development,” and “small molecule catabolic process” showed the highest enrichment. In the Kyoto Encyclopedia of Genes and Genomes (KEGG) enrichment analysis, the “RAP1 signaling pathway” was the most significantly enriched pathway. In addition, KEGG also revealed pathways closely related to angiogenesis, vascular biological activity, and vascular endothelial cell apoptosis, such as “Endococytosis,” “Sphingolipid signaling pathway,” “Cellular senescence,” and “Vascular smooth muscle contraction.” The differential expression of target genes at the differentially methylated sites was calculated, and standardized statistics were performed to draw the expression profile of target genes at these sites in adult patients with ischemic MMD. The volcano map showed that the majority of the target genes did not show significant differences in expression; although, 256 downregulated genes and 758 upregulated genes showed more than double differences in expression compared to that of the control group (Figure 2(c)). Among them, KCMNA1 and SOX6 were the target genes with the highest degree of upregulation and downregulation (CG23291158 and CG00853216), respectively, and they were also among the top 10 target genes with the highest differential expression multiple. Threshold and final differential standard values were set for determining the differential expression multiple of target genes, which will lead to detection errors for genes with high basal expression. After data normalization and adjustment, significant difference in the clustering degree of various methylation sites on the whole was not observed. As shown in the heat map, the expression intensity of CpG sites in 20 samples was uniform. Our results indicated that the differentially hypermethylated sites were mainly distributed in adult ischemic MMD samples. (Figure 2(d)). In addition, large differences in the results of multiple tests may yield false positives in the final results. Therefore, to investigate whether the upregulated and downregulated genes with the highest differential expression ratios can be candidate biological markers for MMD, we continued to perform subsequent validation tests. In addition, the specific biological effects of the abnormal expression of target genes at different methylation sites were verified in vitro.

### 2.4. Highly Enriched Regions of DMPs

Using regression analysis of the significance of the extent of CpG site methylation, we further screened and compared 759 differential methylation probes (adj. *P* value <0.01, ∣ deltaBeta  | >0.2). Most of the DMPs (67.46%) were hypermethylated in patients with MMD (512 hypermethylated and 247 hypomethylated). Overall, the distribution and significance of DMPs differed significantly compared to those of all other probes (*P* < 0.01).

### 2.5. Construction of Cell Lines with Different Expressions of Candidate Genes

**Figure 3:**
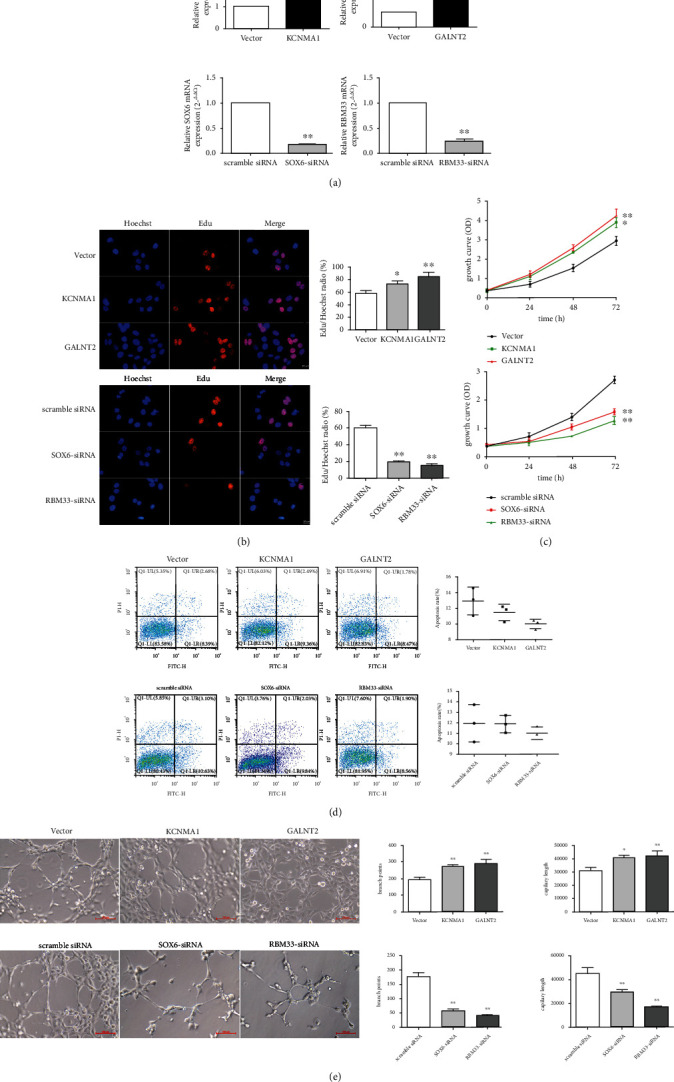
(a) Expression of KCNMA1, GALNT2, SOX6, and RBM33 in HBMECs were determined using RT-qPCR. The results are presented as mean ± SD (*n* = 3). ^∗∗^*P* < 0.01. (b) EdU assay of HBMECs. The proliferating nuclei were stained red with EdU and blue with Hoechst for 2 h. Three random pictures per group were used to count the numbers of positive cells and Hoechst-positive cells. Data are shown as the mean ± SD from three independent experiments (*n* = 3). ^∗^*P* < 0.05, ^∗∗^*P* < 0.01. (c) Growth of HBMECs after the overexpression of KCNMA1 or GALNT2 and knocking down of SOX6 or RBM33. Cell viability was measured using the CCK8 assay after 24 h, 48 h, and 72 h incubation. The results were presented as mean ± SD (*n* = 3). ^∗^*P* < 0.05, ^∗∗^*P* < 0.01. (d) Apoptosis in HBMECs was assessed using flow cytometry after staining with AnnexinV and PI (a). Data are shown as the mean ± SD from three independent experiments. (e) Capillary formation was measured using the tube formation assay. HBMECs from each group were seeded on Matrigel-coated wells and incubated for 72 h to allow the formation of capillary-like structures (*n* = 3). The quantitative data of tube formation assay (b, c). ^∗∗^*P* < 0.01.

As shown in Figure 3(a), The SOX6 mRNA expression decreased significantly in the SOX6-siRNA and rBM33-siRNA groups compared to that in the scrambled siRNA group. These results indicated that cell lines with SOX6 and RBM33 knockdown had been constructed successfully. The KCNMA1 mRNA expression was significantly higher in the KCNMA1 group than in the vector alone group, whereas GALNT2 mRNA level increased significantly in the GALNT2 group. These results indicated that KCNMA1 and GALNT2 overexpression cell lines had been successfully constructed.

### 2.6. Effects of Candidate Genes on Cell Proliferation Capacity and Rate

As shown in Figure 3(b), the EdU staining experiment showed that the proliferation of cells in the KCNMA1 (73.32% ± 4.33%) and GALNT2 groups (84.55% ± 7.26%) were significantly higher than that in the vector control group (57.77%% ± 4.55%). We speculated that the overexpression of KCNMA1 or GALNT2 may increase cell proliferation. Compared to that in the scrambled siRNA group (60.72% ± 2.68%), cell proliferation was significantly reduced in the SOX6-siRNA (19.50% ± 1.05%) and rBM33-siRNA groups (15.44% ± 1.41%), suggesting that SOX6 or RBM33 knockdown can reduce cell proliferation.  Figure 3(c) shows that the overexpression of KCNMA1 or GALNT2 can increase, whereas knockdown of SOX6 or RBM33 can reduce the cell proliferation rate. The detailed data for 0 h, 24 h, 48 h, and 72 h of CCK8 experiment are shown in Table S2 and S3. As shown in Figure 3(d), apoptosis did not differ significantly among the groups. The average and standard deviation of scramble siRNA, sox6-siRNA, and rBM33-siRNA groups were 11.95 ± 1.77, 11.88 ± 0.83, and 11.01 ± 0.61, respectively. And the vector control group, KCNMA1 overexpression group, and GALNT2 overexpression group were 12.93 ± 1.77, 11.45 ± 1.04, and 10.01 ± 0.62, respectively.

### 2.7. Experimental Study on Tubule Formation of HBMEC by Candidate Genes

To elucidate the functional role of the upregulated and downregulated genes in angiogenesis, tubule formation assay was performed on treated HBMECs. As shown in Figure 3(e), the length of the tubules and branch points in the KCNMA1 and GALNT2 groups were significantly higher than that in the vector control. Compared to that in the vector group, the branch points in the KCNMA1 and GALNT2 overexpression groups increased by 39.8% ± 3.6% on average. The average increase in branching points was 49.9.2% ± 7.7%. The tubule lengths in the KCNMA1 and GALNT2 overexpression groups increased by 32.6% ± 4.7% and 37.3% ± 9.0%, respectively. These results suggested that the overexpression of KCNMA1 or GALNT2 can promote tubule formation.

Compared to that in the scrambled siRNA group, the branch points in the SOX6 knocked down cell lines decreased by 67.6% ± 10.4% on average, while those in rBM33 decreased by 77.2% ± 9.3% on average. SOX6 and RBM33 knockdown decreased the tube length by 33.5% ± 5.7% and 62.4% ± 5.6%, respectively. The length of the formed tubule and branch points was significantly lower in the SOX6-siRNA and rBM33-siRNA groups than that in the scrambled siRNA group, suggesting that SOX6 or RBM33 knockdown can inhibit tubule formation.

## 3. Discussion

The pathogenesis of MMD is still poorly understood, and reliable pathogenic factors of this disease have not been determined so far. Only few studies have investigated the epigenetics of MMD. So far, this is the first study to provide DNA methylation profiles of adults with ischemic MMD and without any underlying diseases using 850 K microarray based on genome-wide analysis and to validate target genes and pathways using various in vitro experiments. We observed that the differential methylation sites in some adult patients with ischemic MMD and the control were closely associated with pathways affecting angiogenesis and growth. Some gene loci and their corresponding target genes have the potential to be used as biomarkers of ischemic MMD.

To explain the various complex mechanisms in the pathogenesis of MMD, early studies first focused on differences in gene mutation between regions and ethnic groups affected by MMD (9, 10). The results of these studies support the evidence that genetic factors are the important basis of MMD. Using high-throughput sequencing technology, several studies have investigated the gene loci associated with MMD. This also means that genes with low mutation rates in the population are no longer the only biological markers of MMD (3, 11). Further study of loci of interest requires deep mining and screening of sequencing data. The frequency of occurrence of base pair mutations in the population is low (12). Epigenetic studies on methylation levels (13) revealed differential expression of multiple gene loci, which can be analyzed via stratification for further screening.

In a previous study using endothelial colony-forming cells from 3 patients with moyoya disease, the sortilin1 overexpression modulates the expression of major angiogenic factors and matrix metalloproteinase-9, and the gene can be used as a potential biomarker. (8). Some studies have indicated the importance of investigating the effect of pathways of interest on the occurrence and development of MMD (14–16). However, information regarding the significantly different methylation sites in adult ischemic MMD remains scarce. Furthermore, for the pathways of interest that are affected by the differentially methylated sites, only few comprehensive validation experiments have been performed to verify the results. In this context, this study found that methylation site signals in adult patients with ischemic MMD were generally higher than those in normal controls. The number of differentially methylated sites was the least in the island region and the most in genomic regions, including the promoter region. Target genes significantly upregulated or downregulated at different methylation sites, as well as the influence of some pathways, may eventually lead to the occurrence and development of ischemic moyamoya disease, which needs further experimental verification.

In genome-wide association analysis, we found that after eliminating differences due to race, sex, and biological samples, the results of our analysis showed good pleiotropy, which may lead to the development of varying ischemic MMD symptoms and pathological manifestations. At the same time, we also found that among the genes showing significant differential expression, the number of significantly upregulated genes was higher than the number of significantly downregulated genes (758 vs. 256); however, in general, the number of downregulated genes was lesser than that of the upregulated genes. Finally, we selected the most significant gene body function, “multicellular organismal homeostasis,” and the biological pathway, “RAP1 signaling pathway.” In addition, we also screened the genes, KCNMA1 and GALNT2, which were associated with the differentially methylated sites, and SOX6 and RBM33, which were associated with the highest degree of upregulation. The RAP1 signaling pathway is a small-G protein-mediated signaling pathway that induces a cascade of signals by integrating multiple signals into the endothelium, resulting in a series of cellular responses, including regulation of endothelial barrier function, endocytosis, exocytosis, and neurotransmitter release (17). These studies indicated that the RAP1 signaling pathway plays a key role in regulating endothelial development and function, and that perturbation of this biological pathway may affect the function and growth of vascular endothelial cells (18, 19).

KCNMA1, located at 10q22, encodes and the pore-forming *α*-subunit of the large-conductance Ca^2+^-activated K^+^ channel (20). It also controls the large conducting ion channel and a subunit of voltage activating ion channel (21). Studies have shown that the abnormal function of this gene leads to acquired variation of nervous system function, which possibly leads to movement disorders and epilepsy. In addition, studies have shown that abnormalities in KCNMA1 function can lead to arterial involvement and delayed neurological development (22). At present, relevant gene analysis for patients with MMD is not available. We are the first to observe that in this patient population, KCNMA1 is upregulated by methylation. KCNMA1 promoted cell proliferation but did not affect apoptosis. We used plasmid transfection to overexpress KCNMA1 in HBMECs and observed for the first time that both branch point and tube length had increased.

KCNMA1 edits and regulates the K matriculation (BK_Ca_) pathway. Results from an experiment that activated the BK pathway and simulated rat cerebral vascular smooth muscle cells suggest that the BK_Ca_ channel may be a key in arterial structure remodeling (23). This suggests that the overexpression of this gene is involved in the formation and remodeling of the moyamoya blood vessels in MMD. In addition, other studies have suggested that KCNMA1-encoded cardiac BK channels provide protection against ischemia-reperfusion injury (24).

GALNT2 has previously been used as a regulator of adipogenesis and adipocyte insulin signaling (25). Loss of GALNT2 function may cause novel O-linked glycosylation-related congenital diseases. Maldevelopment and neurodevelopmental abnormalities were observed in GALNT2 knockout rodent models (26). An association between this gene and lipid metabolism has been observed in the carotid intima in an Asian population (27). Downregulation of GALNT2 was involved in abnormal glucose metabolism in diabetes (28). In this study, we found that the overexpressed GALNT2 promoted cell proliferation and tubule formation. Relevant models should be developed to understand whether the metabolic pathway affected by GALNT2 upregulation is involved in the angiogenesis of MMD.

The SOX subfamily of genes is expressed during embryogenesis in different organs of various cell types and has been involved in determining cell fate, proliferation, survival, differentiation, and terminal maturation in many cell lineages (29). Studies in mice reported that systemic deletion of SOX6 resulted in death after two weeks of birth due to premature heart mass and ultrastructural changes in the heart and skeletal muscle (30). SOX6 has not been reported in MMD; however, a study indicated that SOX6 may inhibit the proliferation and invasion of ovarian cancer cells and tumor cell-induced angiogenesis (31). In addition, miR-499 inhibits hypoxia/reoxygenation-induced cardiomyocyte apoptosis by downregulating SOX6 (32). These studies highlight the importance of SOX6, and results of our cell proliferation experiments are consistent with those of the above studies. For the first time, we used HBMECs to observe tubule formation after down-regulation of SOX6, suggesting that this gene is involved in the development of human microvascular endothelium.

RBM33 induces the signaling network of inflammatory response in host immune cells and participates in the immunomodulatory response to lymphatic filariasis (33). Reports show that RBM33 promotes the progression of gastric cancer by targeting miR-149 to regulate IL-6, and that the circular RNA of RBM33 is involved in the progression of cervical cancer by regulating the miR-758-3p/PUM2 axis (34, 35). Few in vitro studies on this gene are currently available. Studies on apoptosis of individual peripheral blood monocytes showed that the reduced IL-10 expression in RBM33 stimulated the major Th1 response in THP-1 monocytes, which was antigen-specific, but RBM33 did not induce apoptosis of either monocytes or lymphocytes (36). We also found that RBM33 did not affect apoptosis.

This study has some limitations. Interpretation of our results may be limited by the sample size of the current chip analysis. In addition, in the absence of successful out-of-body model of MMD, the four target genes designed in this study were only verified from the perspective of human microvascular endothelial cells. Nevertheless, these results suggest the role of the whole epigenome in identifying the underlying pathogenesis of MMD.

## 4. Conclusion

Currently, studies that elucidate the role of DNA methylation profiles and abnormal expression of the above genes in human brain microvascular endothelial cells are lacking. Our study shows the importance of DNA methylation in the development of adult ischemic MMD. In addition, identification of some genes with significantly different methylated sites can be an important research direction for investigating the biological markers of MMD, which will be critical for guiding the diagnosis and screening of this disease. The effect of the regulated genes on tubule formation in HBMECs also helped in explaining the phenomenon of cerebrovascular occlusion and smoky blood vessel formation in MMD patients. Next, we will further model these newly discovered methylation regulated susceptibility genes.

## 5. Materials and Methods

### 5.1. Study Design and Inclusion and Exclusion Criteria

The whole blood samples used in this study were obtained from patients admitted to Beijing Tiantan Hospital from September 2020 to July 2021. Ten adult patients, all Chinese and Han nationality, were evaluated for ischemic moyamoya disease by digital subtraction angiography (DSA). After detailed consultation and physical examination, all participants were found not to have any underlying diseases that could affect the results of the study, such as hypertension, diabetes, hyperlipidemia, hyperthyroidism, and surgical history. In addition, 10 age and gender-matched healthy subjects were included in this study.

### 5.2. DNA Extraction from Experimental Specimens

The Gentra Puregene DNA extraction kit (Qiagen, Venlo, Netherland) was used for extracting DNA from whole blood samples. The extracted samples were modified with sulfites using the EZ DNA kit (Zymo Research, Irvine, CA, USA). The DNA samples were prepared for amplification and array hybridization, which included DNA fragmentation and DNA precipitation. The ratio of the absorbances at 260 nm and 280 nm of all samples ranged from 1.7 to 2.0, the concentration was ≥50 ng/*μ*L, and the total amount was ≥2 *μ*g (37). All samples were dissolved in Tris-EDTA before preparation and were stored in a -80°C Forma 700 ultra-low temperature refrigerator (Thermo Ltd.).

### 5.3. Illumina 850K Chip Hybridization and Data Quality Control

The Illumina Infinium MethylationEpic BeadChip was used for hybridization with the extracted DNA fragments. After chip cleaning, single base extension, and staining, the Illumina ISCAN system was used for chip scanning. During data scanning and input, the 850 K chip performs the necessary quality control for staining and extension for each sample to ensure that the experimental procedures and the chip are used correctly.

In this study, the quality of the raw data was strictly controlled to ensure the accuracy of the epigenomic association analysis (EWAS). Based on the base reaction characteristics of two types of probes, Infinium I and Infinium II, on the 850 K chip, the *P* values of detecting the signal of each probe can be selected (*P* < 0.01). Multiple magnetic beads distributed on different probes reflect the fluorescence signal strength (bead value) collected, and samples and sites can be screened according to the signal strength (bead value ≥ 3). Single nucleotide polymorphisms (SNPs) interfere with the quality of original data and are not beneficial for EWAS analysis for single disease; hence, we used partial quality control probes to exclude these sites (38). The “ChAMP” package was used to perform intergroup quality control of probe data, and different chromosome regions detected using the same probe were excluded. Recent epidemiological studies on MMD have shown that the incidence of MMD does not vary with gender (39). Therefore, in this study, CpG sites on sex chromosomes were filtered out. In addition, the loci that differed negligibly among the samples were also deleted. In the end, 726,339 probes (853,307 probes in total) from 20 samples were included in the dataset for this study.

### 5.4. Methylation Site Association Analysis

The original data of all samples in the platform were imported into *R* (3.6.0; https://cran.r-project.org/), tidyr, tibble, and dplyr for batch processing of the original data. The ChAMP package was used for intergroup comparisons of data. The limma package was used to partition and preprocess the difference data. The independent variable adopted in this study was the preprocessed beta matrix of methylation level. Considering that the methylation level represented by beta follows the binomial distribution based on multiple tests, the beta matrix to be processed was statistically tested by using the “Limma” package, in order to describe the regularity of discrete random events whether a certain point is a significant methylation site. According to the data preprocessing results, values with significant differences between groups (*P* < 0.05) and the corrected sites were classified as methylation sites. Similarly, cluster data of differential methylation sites ranging from several hundred base pairs to several mega bases were classified as differentially methylated regions (DMRs) after multiple corrections (40).

### 5.5. Selection of Focus Genes and the Study of Hot Spot Pathways

The genetic information of the existing hot spots of MMD genes and their regulatory pathways were screened to identify more potential epigenetic biomarkers for MMD. Based on the gene name and region corresponding to the identified gene loci, we annotated part of the unexplored loci using bioconductor. The replicable candidate sites with the largest difference level were set as alternatives to further screen the original genes with DMPs for verification of significance and improve the collective predictive ability of the candidate genes. The candidate genes were screened based on the adjusted *P* values of logFc and information regarding DMPs of all probes. After the relevant original genes were identified, PubMed and other online databases were used to search the progress in research regarding the relevant genes and hotspot pathways. This allowed refinement of the scope for further studies on the regulatory mechanisms of genes and pathways.

### 5.6. Bioinformatic Analysis

The original data of the MMD patient and the healthy control groups were sampled using the limma package. The logarithmic difference in the gene expression was calculated after further statistical normalization. Return of the logFC value represented the width of the expression. Differences in the expression levels of all genes between patients with MMD and healthy controls are represented by the logFC of all probes and the adjusted *P* value obtained using the unpaired *t*-test. The gene loci and the original genes were annotated, and their interactions were predicted using GPL-related search interface in the GEO database. The Gene Ontology database (GO, http://david.abcc.ncifcrf.gov/) was used for predicting gene function. The Kyoto Encyclopedia of Genes and Genomes (kegg, http://www.genome.ad.jp/kegg/) was used for gene pathway analysis.

### 5.7. In Vitro Validation and Experimental Cell Preparation

Human brain microvascular endothelial cells (HBMECs) from ScienCell Research Laboratories (CA, USA) were used for in vitro validation experiments. All cells were cultured in endothelial cell culture medium for 10-15 generations to eliminate the effects of cell growth factors produced by naive protocells as much as possible. All media were composed of 500 mL basal nutrients, 25 mL fetal bovine serum, 5 mL endothelial cell growth factors, and 5 mL penicillin solution (41). When the cells were more than 90% confluent, the medium was replaced, and the cells were washed several times with 2 mL phosphate buffered saline (PBS) to eliminate the residual cell growth factors. After rinsing the buffer with normal saline, 2 mL of 1 : 250 diluted trypsin (0.25% (w/v) trypsin-0.53 mM EDTA) was added. The process of dissociation was observed under a microscope; when the contact between the cells was significantly reduced, the digestion was terminated, followed by the addition of 2 mL complete medium to stabilize the cells. After the excess water on the cell surface was dried naturally, the cells were collected while being shaken slightly. The collected cultured cells were centrifuged at 800 rpm at 4°C for 5 min. After centrifugation, the supernatant was removed, and the cells were placed in complete medium and cultured in bottles. The medium was replaced every two days.

### 5.8. Small Interfering RNA (siRNA) Transfection and Experimental Cell Grouping

The siRNAs used for transfection were divided into three groups: scrambled siRNA, SOX6-siRNA, and RBM33-siRNA (Table 3). The following siRNA transfection steps were completed in each group, and the subsequent experiments were performed 48 h after transfection. All cultured cells used for siRNA transfection procedures were microscopically confirmed to be in the logarithmic growth phase 24 h before transfection. The HBMEC culture in logarithmic growth phase was dissociated with trypsin mixed with digestive fluid. The isolated cultured cells (0.5 × 10^5^) were incubated in 10% cell medium in the absence of penicillin. The cells were reinoculated in 24-well plates and cultured at 37°C in the presence of 5% CO_2_ till they were 70-80% confluent. All cultured cells were placed in serum-free medium 2 h before transfection. Reagent A was prepared by dissolving 20 pmol siRNA in 50 *μ*L Opti-MEM serum-free medium. Reagent B was prepared by dissolving 1 *μ*L LiPO2000 in 50 *μ*L Opti-MEM serum-free medium and mixing at room temperature for 5 min. The serum-free medium for siRNA transfection consisted of a mixture of reagents A and B. Reagent A was prepared by dissolving 20 pmol siRNA in 50 *μ*L Opti-MEM serum-free medium. Reagent B was prepared by dissolving 1 *μ*L LiPO2000 in 50 *μ*L Opti-MEM serum-free medium and mixing at room temperature for 5 min. Reagents A and B were mixed and incubated for 20 min. The mixed reagent (400 *μ*L per well) was added to the corresponding 24-well plate, which was replaced with serum containing colorectal cell culture medium after 6 h (41).

### 5.9. Quantitative Reverse Transcription-Polymerase Chain Reaction (qRT-PCR)

First, RNA was extracted from cells in each group (same as that in the siRNA transfection experiment). Cell suspensions were prepared using 0.25% (w/v) trypsin-0.53 mM EDTA, and 100,000 cells were inoculated into each well of different 6-well plates. The supernatant was removed when the cells were 90% confluent, followed by the addition of 1 mL TRIzol Reagent (Takara, Japan) to each well. The plates were shaken mildly, transferred to Eppendorf tubes, and allowed to stand for 5 min. Then, 0.2 mL chloroform was added to each well and shaken violently. After standing for 10 min, the tubes were centrifuged at 12,000 rpm at 4°C for 15 min. The supernatant was mixed with 400 *μ*L isopropyl alcohol, mixed via violent agitation, and incubated at -20°C for 30 min. After the supernatant was discarded, the pellet was washed with 1 mL of 75% ethanol by centrifuging at 7,500 rpm and 4°C for 5 min. After discarding the supernatant, the pellet was air-dried for 5-10 min. The final precipitated RNA was dissolved in 10 *μ*L diethyl pyrophosphate (DEPC) (Amresco, Ohio, USA) (8). Two microliters RNA solution was used to determine the concentration and purity of the final extracted mRNA using a Nanodrop 2000 spectrophotometer (Thermo Fisher).

One microgram RNA, 1 *μ*L of 500 *μ*g/mL thymine nucleotide chain, and 10 *μ*L DEPC solution were mixed. The reverse transcriptional reaction was performed at 70°C for 5 min, and the reaction products were removed and placed on ice for 5 min. After cooling, 10 *μ*L of the product was added to a solution containing 4 *μ*L buffer, 2 *μ*L dNTP, 1 *μ*L reverse transcriptase, and 0.5 *μ*L RNA inhibitor; the final volume was made up to 20 *μ*L with DEPC solution. The reaction was performed as follows: 20°C for 5 min, then 42°C for 1 h, 70°C for 15 min, and finally cooled to 4°C for over 2 min. After cooling, the cDNA was harvested and stored at -20°C. One microliter cDNA was added to an enzyme-free 200 *μ*L microcentrifuge tube to which were added 7.4 *μ*L nuclease-free water, 10 *μ*L Ssofast Evagreen super mix (Bio_Rad), 0.8 *μ*L each of forward, and reverse primers in a total volume of 20 *μ*L for qRT-PCR. The tubes were sealed and placed in the LightCycler 480 real-time PCR system (Roche Applied Science). The reaction parameters were set for amplification, and the fold change in expression was calculated using the 2^-*ΔΔ*Ct^ method. The PCR primers used were from Sangon Biotech (Shanghai, China). The sequences and names of the primers are shown in Table 4. The sequence, cell preparation, plasmid identification, amplification, extraction, and transfection of KCNMA1 and GalNT2 genes can be found in the Supplemental Data.

### 5.10. Cell Proliferation Assay (CCK8)

In total, 2,000 HBMECs in logarithmic growth phase were seeded in 96-well plates and cultured at 37°C in the presence of 5% CO_2_ for 12 h. After the cells adhered to the wall, 10 *μ*L CCK8 solution was added to each well and incubated for 4 h, 24 h, 48 h, and 72 h. Absorbance was determined at 450 nm using the Berthold LB941 microplate multifunctional enzyme plate analyzer (Berthold Company) after incubation for 4 h at each time node.

### 5.11. EdU Cell Proliferation Assay

The grouping of cells used in the EdU experiment was the same as that in the siRNA experiment. Cells in each group were seeded in 96-well plates at the density of 40,000 cells per unit and incubated for 24 h. The EdU was dissolved and diluted 1000 : 1 in basic cell medium. 100 *μ*L EdU was added to the medium in each well and incubated for 2 h. Subsequently, the cells were washed twice with PBS for no less than 5 min to remove the excess medium. Then, 50 *μ*L cell fixative solution was added to each well and incubated at room temperature for 30 min, following which the fixative solution was removed. Next, 50 *μ*L of 2 mg/mL glycine was added to each well and incubated for 30 min in a decolorization shaker, after which it was removed. Then, 1× Apollo dyeing reaction solution (100 *μ*L) was added to each well and incubated in a decolorization shaker in a dark room at room temperature for 30 min.

The dyeing reaction solution was removed, followed by the addition of 100 *μ*L penetrant and washing thrice in the decolorization shaker for not less than 10 min each time. The penetrant was removed after cleaning. The reagent was then diluted 100 : 1 in deionized water. Fluorescent dye 1× Hoechst33342 was added and stored away from light. Then, 100 *μ*L 1 × Hoechst33342 reaction solution was added to each well, and the plates were incubated with decolorization shaker in the dark at room temperature for 30 min. After incubation, the staining solution was removed, and the cells were washed thrice using 100 *μ*L PBS. After washing, the staining solution was detected immediately using a Zeiss LSM laser confocal microscope (Zeiss GMBH).

### 5.12. Flow Cytometry

The AnnexInv_PI double dyeing kit (Invitrogen, California, USA) was used for flow cytometry. The experimental grouping was the same as before. An appropriate amount of 1× Annexin binding solution was prepared with 100 *μ*g/mL propidium iodide dye (PI working solution). To detect apoptosis in each group after a series of cell proliferation experiments in vitro, the cells were continuously cultured in the basic medium for 24 h. After culturing, the cells were collected and washed in PBS precooled at 4°C. After cleaning, the supernatant was centrifuged at 4°C, 1,000 rpm, for 5 min, and the supernatant was removed after centrifugation. 1× Annexin binding solution was used to resuspend the cells, and the cell density was adjusted to 100,000 cells/mL. After adjustment, 5 *μ*L Alexa Fluor 488 Annexin V and 1 *μ*L 100 *μ*g/mL PI solution were added to each 100 *μ*L cell suspension (42). Finally, the cells were cultured at room temperature for 15 min. After incubation, apoptosis was analyzed in vitro using a DXFlex flow cytometer (Beckman Coulter) (43).

### 5.13. Tubule Formation Assay to Verify Cell Growth In Vitro

This includes preparation of organ-like culture medium, implantation of grouped cells, and collection and analysis of tubular structures. Preparation of organ-like medium: Matrigel glue stored at -20°C was transferred to a refrigerator at 4°C for thawing 24 h before the experiment. The pipetting gun head was kept in a refrigerator at -20°C for precooling. The spear head was taken out and put on ice for use 30 min before the experiment. Next, 50 *μ*L of overnight melted Matrigel glue was added per well to a 96-well plate with slight shaking to avoid bubble formation and incubated at 37°C for 30-60 min to solidify the Matrigel glue. Cell implantation: HBMECs in each group were routinely digested and centrifuged and then resuspended in basic medium to obtain cell density of 200,000 cells/mL. Then, 100 *μ*L single-cell suspension was slowly added along the wall into the well without touching the glue surface. Three duplicate wells were used for each sample, and the 96-well plate was incubated at 37°C for 72 h. Collection and analysis of tube formation: the lumen was completely formed after 72 h of cultivation. The 96-well plate was removed and observed under a microscope. The radiographs were crosschecked by two skilled operators. Each experiment was repeated thrice. The ImageJ (https://imagej.nih.gov/ij/) software was used to analyze the length of each group of cells forming the branching lumens and the results were averaged.

## Figures and Tables

**Table 1:** Characteristics and clinical information of participants.

Characteristics	MMD group (*n* = 10)	Control group (*n* = 10)	*P* value
Mean, age ± SD (years)	38.3 ± 10.5	33.9 ± 8.4	0.34
Male	5 (50%)	5 (50%)	1
Female	5 (50%)	5 (50%)	1
Diseased cerebral hemisphere			
Left	3 (33.3%)		
Right	3 (33.3%)
Double	4 (40.0%)
Mean duration of symptoms ± SD, (years)	2.1 ± 2.5
Suzuki stage (mainly the high-level side)			
1	1 (10%)		
2	2 (20%)
3	1 (10%)
4	1 (10%)
5	3 (30%)
6	2 (20%)

SD: standard deviation.

**Table 2:** The top ten CpG with the highest differential expression.

DMP	*P* value	logFC (abs)	Regulation	Gene	Feature	Cgi
cg00853216	3.121069*e*-10	0.7029178	Down	SOX6	5′UTR	Opensea
cg13295089	4.770167*e*-09	0.6909873	Down	RBM33	Body	Opensea
cg18213661	5.833473*e*-10	0.5843512	Down	IGR	Opensea	IGR-opensea
cg16791832	4.552424*e*-09	0.5772866	Down	IGR	Shore	IGR-shore
cg00513811	9.058064*e*-07	0.5688403	Down	MYO1D	Body	Opensea
cg23291158	2.858392*e*-08	0.5659156	Up	KCNMA1	Body	Opensea
cg14044167	7.786136*e*-10	0.5516543	Down	NDRG1	Body	Opensea
cg26303777	1.388451*e*-07	0.5504684	Up	GALNT2	Body	Opensea
cg27273227	1.532697*e*-06	0.5465678	Down	IGR	Opensea	IGR-opensea
cg01886237	1.946201*e*-07	0.5412209	Down	IGR	Opensea	IGR-opensea

**Table 3:** Sequence of SOX6 and RBM33.

siRNA	Sequence
SOX6-siRNA	CCAGCCCTGTAACTCAAGTTA
RBM33-siRNA scrambled siRNA	GCAGAAATTCCACAGGCATAT nonfunctional sequence

Scrambled siRNA: a nonfunctional sequence of small infective RNA was used as a control to verify the possible inhibitory effects of siRNA.

**Table 4:** Primers used in quantitative real-time PCR.

Primers	Sequence (5′ →3′)	
SOX6	Forward	CACATGACAGCCTCAGAGCA
	Reverse	ATTCCCCTCCTTCTCCTCCC
RBM33	Forward	GGACAGTATGAAGGCCACGA
	Reverse	GTTTCCATACCTCCTGTAAATTCAT
GAPDH	Forward	CATGTTCGTCATGGGGTGAACCA
	Reverse	AGTGATGGCATGGACTGTGGTCAT

## Data Availability

All data generated or analyzed during this study are included in this published article. Some or all data, models, or code generated or used during the study are available from the corresponding author by request.

## Abbreviations

MMD: Moyamoya disease
GO: Gene Ontology
KEGG: Kyoto Encyclopedia of Genes and Genomes
qRT-PCR: Quantitative reverse transcription-polymerase chain reaction
EWAS: Epigenomic association analysis
SNPs: Single nucleotide polymorphisms
PBS: Phosphate buffered saline
CCK8: Cell proliferation assay.

## Supplementary Materials

Supplementary Materials includes cell preparation to verify significantly upregulated genes (KCNMA1 and GALNT2), plasmid identification, amplification, extraction and transfection, and detailed results of CCK8 assay. Table S1: primers used for qRT-PCR. Figure S1: target gene construction. Table S2: results of CCK8 experiment in KCNMA1 and GALNT2 overexpression group. Table S3: results of CCK8 experiment in SOX6-siRNA and rBM33-siRNA groups.
