# Supplementary material for: Epigenome-Wide Association Study Reveals Differential Methylation Sites and Association of Gene Expression Regulation with Ischemic Moyamoya Disease in Adults
Source: Oxid Med Cell Longev. 2022 Mar 24;2022:7192060. doi: 10.1155/2022/7192060 (PMC8970806; doi:10.1155/2022/7192060)
Supplement: Supplementary Materials — includes cell preparation to verify significantly upregulated genes (KCNMA1 and GALNT2), plasmid identification, amplification, extraction and transfection, and detailed results of CCK8 assay. Table S1: primers used for qRT-PCR. Figure S1: target gene construction. Table S2: results of CCK8 experiment in KCNMA1 and GALNT2 overexpression group. Table S3: results of CCK8 experiment in SOX6-siRNA and rBM33-siRNA groups. [file 7192060.f1.pdf]

Supplementary Information

Epigenome-wide reveals differential methylation sites and association of gene expression regulation in moyamoya disease

Shihao He*,[1] MD; Ran Duan*,[3] MD; Yahui Zhao,[1] MD; Yanchang Wei,[1] MD; Ziqi Liu,[1] MD; Xiaolin Chen,[1] MD; Xun Ye[1, 3] MD; Qiang Hao,[1] MD; Hao Wang,[1] MD; Cunxin Tan,[3] MD; Yu Chen,[1] MD; Junlin Lu,[1] MD; Runting Li,[1] MD; Zhipeng Li,[1] MD; Debin Yan,[1] MD; Haibin Zhang,[1] MD; Fa Lin,[1] MD; Kexin Yuan,[1] MD; Yuanli Zhao[1, 2, 3] MD; Rong Wang[1, 2, 3] MD;

[1]Department of Neurosurgery, Beijing Tiantan Hospital, Capital Medical University, Beijing 10070, China

[2]Center of Stroke, Beijing Institute for Brain Disorders, Beijing 100069, China

[3]Department of Neurosurgery, Peking University International Hospital, Beijing 102206, China

Correspondence to:

Rong Wang, E-mail: ronger090614@ccmu.edu.cn, Telephone: +8613001190333, Fax: 86-10-67096523.

Yuanli Zhao, E-mail: zhaoyuanli@126.com, Telephone: +8613801121203, Fax: 86-10-67096523.

Supplementary Materials and Methods

Preparation and grouping of experimental cells

To verify the effect of significantly up-regulated genes (*KCNMA1* and *GALNT2*) on cell proliferation and development during the expression process, the following groups were set: no-load group: unbound pure plasmid was transfected into HBMECs for reserve use; *KCNMA1* overexpression group: the *KCNMA1* sequence in the overexpression vector was transfected into HBMECs, which was cultured for 48 h for later use; *GALNT2* overexpression

group: the *GALNT2* sequence in the overexpression vector was transfected into HBMECs, which was cultured for 48 h for later use.

All the overexpressed vectors were prepared according to laboratory standards, and the target gene was amplified according to the design of the transfection primers.

Table S1. Primers used for qRT-PCR

Primer	Sequence (5′→3′)
KCNMA1-F	CTAGCTAGC ATGGCAAATGGTGGCGGCG
KCNMA1-R	GATCTCGAG TCATCTGTAAACCATTTCTTTTCTG
GALNT2-F	CTAGCTAGC ATGGCTGACAGAGAGGACTGG
GALNT2-R	GATCTCGAG CTACTGCTGCAGGTTGAGCG

Figure S1. Target gene construction

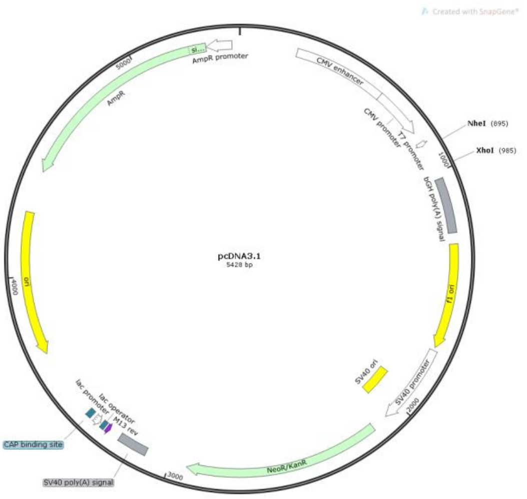

After determining the structure of the transfection vector and the sequence to be transfected, the following were mixed in a total volume of 50 µL for restriction enzyme digestion: ddH_2O, 41 µL; 10× CutSmart Buffer2, 5 µL; 1 µg/µL pure plasmid DNA; 1 µL

Nhe I (10 U/μL) and 1 μL Xho I (10 U/μL). The solution was mixed via mild agitation, centrifuged at 3,000 rpm for 1 min, and incubated at 37 °C for 3 h. Agarose gel electrophoresis was performed on the digestion products of the carrier, and the target bands were recovered. One microliter of the linearized vector (100 ng/μL) was mixed with 1 μL double-stranded DNA (100 ng/μL), 2 μL ligase buffer, and 20 μL ddH_2O to set up the ligation reaction overnightat 16 °C. After the reaction, various ligated products were added to 100 μL of different groups of experimental cells, mixed gently, placed on ice, and incubated for 30 min. Then, the cells were heated at 42 °C for 90 s and incubated in ice-water for 2 min. After the culture, 500 μL basic medium was added and the cells were shaken at 37 °C for 1 h. An appropriate amount of bacterial liquid was evenly spread on a plate containing ampicillin and incubated for 16 h.

Identification, amplification, extraction, and transfection of plasmids

First, PCR was performed for identification of genetic material in a colony, followed by extraction of plasmid from a PCR-positive colony, and transfection of the plasmid in experimental cells. The PCR system of 20 μL consisted of 10 μL 2× Taq Plus master mix, 9.2 μL ddH_2O, and 0.4 μL each of upstream downstream primers. The reaction system was mixed via mild agitation and centrifuged at 3,000 rpm for 1 min. Next, a single colony was selected and placed in the reaction system using the head of a sterile gun on a sterile working table, mixed evenly, and placed in a PCR instrument. The following reaction parameters (DNA denaturation: at 94 °C for 3 min; annealing (22 cycles) at 94 °C for 30 s, 55 °C for 30 s, 72 °C for 30 s; elongation at 72 °C for 5 min; cooling at 4 °C) were used. The colonies identified as positive clones were inoculated in liquid medium containing ampicillin and cultured at 37 °C for 16 h. After the completion of culture, an appropriate amount of bacterial liquid was taken for sequencing. Bacteria, sequencing results of which were consistent with the experimental grouping, were inoculated in 10 mL basic medium containing ampicillin and cultured overnight at 37 °C. After the culture, the bacterial liquid was collected in different 5 mL centrifuge tubes and centrifuged at 12,000 rpm for 2 min. After centrifugation, the supernatant was removed, 250 μL cell resuspension was added, and the mixture was evenly mixed via sufficient oscillation. Cell lysate (250 μL) and 10 μL proteinase K were mixed via oscillation for 5–6 times and then allowed to stand for 1–2 min. Next, 350 μL buffer solution was added, and the mixture was reversed and shaken again to completely

precipitate the protein product. The solution was incubated in ice water for 5 min. After freezing, the proteins were centrifuged at 10,000 rpm for 10 min. After centrifugation, the protein products were removed and the supernatant was aspirated in another sterile 1.5 mL tube. After centrifugation at 2,000 rpm for 5 min, the supernatant was transferred to a sterile recovery column. After centrifugation at 12,000 rpm for 1 min, the lower waste liquid was removed, and 600 μL of the rinsing liquid was added. The lower waste liquid was removed after centrifugation for 1 min at 12,000 rpm (the residual rinsing liquid was removed again as before). After the rinsing solution was cleaned, the recovery column was transferred to a new 1.5 mL tube on the aseptic operating table and allowed to stand for 10 min. Next, the column was air-dried, followed by addition of nuclease-free water. After standing for 2 min, the column was centrifuged at 12,000 rpm for 2 min and numbered.

Table S2. Results of CCK8 experiment in KCNMA1 and GALNT2 overexpression group

					AVERAGE	SD
Vector control group	0h	0.3456	0.4232	0.3362	0.368333	0.047748
	24h	0.7913	0.7704	0.5401	0.7006	0.139389
	48h	1.454	1.7553	1.4083	1.5392	0.188538
	72h	2.7236	3.1746	2.9519	2.950033	0.225506
KCNMA1 overexpression	0h	0.2982	0.4559	0.3637	0.3726	0.079226
	24h	1.2665	1.0356	1.0337	1.111933	0.133862
	48h	2.3927	2.2852	2.3555	2.344467	0.054593
	72h	3.6188	4.136	3.9454	3.900067	0.261563
GALNT2 overexpression	0h	0.2944	0.3936	0.3494	0.3458	0.049698
	24h	1.0286	1.3957	1.1895	1.2046	0.184015
	48h	2.443	2.6022	2.7359	2.5937	0.146635
	72h	4.2485	4.5985	3.9257	4.257567	0.336492

Table S3. Results of CCK8 experiment in SOX6-siRNA and rBM33-siRNA groups

					AVERAGE	SD
scramble siRNA	0h	0.3618	0.4131	0.3349	0.369933333	0.039729376
	24h	0.7726	0.7647	0.6223	0.719866667	0.084587489
	48h	1.4328	1.467	1.2748	1.391533333	0.102530061
	72h	2.5732	2.8635	2.6783	2.705	0.146980237
SOX6-siRNA	0h	0.3543	0.4276	0.4472	0.4097	0.048968459
	24h	0.5721	0.4851	0.5681	0.541766667	0.04911551
	48h	1.0672	1.1722	0.9197	1.053033333	0.126844721
	72h	1.6194	1.4449	1.6996	1.587966667	0.130226969
RBM33-siRNA	0h	0.3516	0.4029	0.3431	0.365866667	0.032352177
	24h	0.6292	0.4763	0.4337	0.513066667	0.102805172
	48h	0.74	0.7295	0.6915	0.720333333	0.025516335
	72h	1.3683	1.1306	1.3504	1.2831	0.132371787
